# Supplementary material for: Hearing the voices of midwives through reflective writing journals: Qualitative research on an educational intervention for Respectful Maternity Care in Zimbabwe
Source: PLOS Glob Public Health. 2023 Dec 22;3(12):e0002008. doi: 10.1371/journal.pgph.0002008 (PMC10745140; doi:10.1371/journal.pgph.0002008)
Supplement: Attachment — Submitted filename: Response to reviewer 28 6 23.docx [file pgph.0002008.s001.docx]

| **Hearing the voices of midwives through reflective writing journals: qualitative research on an educational intervention for Respectful Maternity Care in Zimbabwe** | |
| --- | --- |
| **Reviewer’s comments** | **Authors’ responses** |
| **Generic comments**  Thank you for the constructive comments made by the reviewer. We have done our best to accommodate them and have given reasons when we could not. We believe the paper is stronger and clearer as a result. | |
| Language- Please don’t refer to women and other people in labour and birth as ‘patients’. Please also change ‘delivery or delivering’ to ‘giving birth’ unless it is in a quote. | We have changed the wording to women in their care, women in labour, birthing women and women, unless in quotes.  The words delivery and delivered were changed to birthing, giving birth or gave birth, except in quotes. |
| Methods- would be good to get more details on the journals, the language, the challenges in analysis etc. | The journals were hand-written in English which is the language they were trained with. Limitations with the content of the journals (such as social desirability bias) have been explored under reflexivity.  The main challenge in analysis was if the midwife did not give enough narrative in her reflection to explain the story. These details usually came through during the group discussions. |
| Positionality- Were there any midwives or nurse-midwives amongst the co-authors? Please include a little more about the co-authors. | Three of the co-authors were midwives, three were doctors and two were health professional educators. The section on the facilitators in the methodology has been extended to include more on the co-authors. |
| ‘Compassion’ and ‘empathy’ have been used interchangeably in the manuscript. Please look into the meaning of and differences in these two and use the concepts accordingly. | We have noted the debate on use of the term empathy in training health professionals and have deleted its use entirely in the text (cf Bloom 2017). It has been replaced with compassion and resilience with definitions given for clarity. |
| **Specific comments** | |
| Page 15- last paragraph- Behaviour change is not as simple as suggestions from peers to smile more and be kind. Please look back into the journals to find more on what makes them loose that smile, loose their compassion and be unkind in the first place. What are the structural and policy related barriers and gender and power related issues that makes RMC challenging in obstetric settings? There is potential to add a sub-section about this in the findings to then understand this better. | In the discussion, paragraph 6, the structural barriers to RMC are acknowledged, as are power and gender related issues in paragraph 3 of the Discussion. However, the study was not designed to assess these. The stated objective of the study was “*to assess changes in attitudes, behaviours and practices in a group of midwives in response to an educational intervention promoting RMC*”. We recognise that it is difficult to change those barriers in under-resourced health systems. Landmark papers have been written promoting transformative learning as the way forward for strengthening health systems (Frenk et al Lancet 2010). We wanted to study whether working with midwives to enhance their agency through transformative learning could change their approach to birthing women in sustainable ways. A year after the intervention the effect of RMC training has persisted.  A paragraph has been added at the end of the results to acknowledge again the importance of structural barriers and a recommendation made for this to be a future research priority.  The recommendation by Frenk et al and reference to WHO on positive birth experiences have been moved to the introduction. |
| Page 16- 1st paragraph about midwives’ newfound confidence and authority- While this has been mentioned in the findings as well. It is not as easy. What made the challenges / barriers that they may have faced before go away? what are the repercussions of them exercising this newfound agency? if they are not understood better then chances are that with time the old team culture will catch up. | The study was not designed to evaluate the repercussions of their enhanced agency beyond the one year as described. The midwives described changes they made as a result to their team culture which persisted a year later, especially bringing RMC issues to their team meetings, running workshops for their colleagues, discussing examples of changing practices so that others were encouraged to do the same.  The reviewer is correct to say it was not easy. Using the transformative learning process to confront their previous attitudes and culture of abuse, the midwives did a lot of deep learning to achieve their personal and professional growth through discussing the incidents they had found traumatic, their developing trust in each other to reveal their previously abusive behaviour and to show how they could be different. The smiling and kindness, along with breathing, singing, sharing experiences, were their methods for coping. As we say in the text, “*Learning experiences are said to be transformative when the changes claimed persist and endure, when they are not limited to behaviours but to a way of seeing the world, being more open-minded, inclusive and having deeper self-awareness”* |
| Last paragraph about uncooperative children- I understand that this language of 'disciplining naughty children' has been referenced to in the introduction as well. But literature suggests that this is also gender -based and there is plenty of evidence of gender-based violence being about disciplining women and obstetric violence is one of it’s kind as well. The literature around women self-disciplining their behaviour and bodies to avoid obstetric violence also points to that. Please bring that into the discussion. | The references to naughty children have been removed.  This paper is written from the perspectives of midwives in the study and the issue of women self-disciplining their behaviour and bodies to avoid obstetric violence did not come up in their discourse.  One example from the journals has been added to show how sexual trauma may lead to difficulties in childbirth. |
| Page 17- 2nd paragraph about midwives’ helplessness- Exactly! More on this in the findings from the midwive's journals please. | More quotes from the journals have been added to show how the midwives turned their helplessness to power, especially in relation to the structural constraints they faced. |
| Maternal morality meetings- It is unclear to me whether these meetings are being attended by just midwives or the entire team of care providers. | The maternal mortality meetings are attended by the whole range of health professionals involved in maternity services for the Greater Harare Maternity Unit. This has been clarified in the text. |
| Page 18- 1st paragraph about future research- Please bring in suggestions that are about changing the team culture and not just focused on the midwives. | Suggestions for future research have been added on this. |
| Conclusion-  Page 19- From what I understand, this study explores this only in the context of midwives not all health care professionals. Please change the sentence. Every category of health care professional's challenges are unique, so it won't be fair to generalize this to health care providers. | Sentence amended as suggested. |
| References- please provide the doi of all the references where applicable. | Provided. |
